# Investigation of the *In Vitro* Therapeutic Efficacy of Nilotinib in Immortalized Human *NF2*-Null Vestibular Schwannoma Cells

**DOI:** 10.1371/journal.pone.0039412

**Published:** 2012-06-20

**Authors:** Nesrin Sabha, Karolyn Au, Sameer Agnihotri, Sanjay Singh, Rupinder Mangat, Abhijit Guha, Gelareh Zadeh

**Affiliations:** 1 Arthur and Sonia Labatt Brain Tumour Research Centre, Hospital for Sick Children Research Institute, University of Toronto, Toronto, Canada; 2 Department of Surgery (Neurosurgery), Western Hospital, University of Toronto, Toronto, Canada; University of Patras, Greece

## Abstract

Vestibular schwannomas (VS) are a common posterior fossa brain tumor, and though benign can cause significant morbidity, particularly loss of hearing, tinnitus, vertigo and facial paralysis. The current treatment options for VS include microsurgical resection, stereotactic radiosurgery or close surveillance monitoring, with each treatment option carrying associated complications and morbidities. Most importantly, none of these options can definitively reverse hearing loss or tinnitus. Identification of a novel medical therapy, through the use of targeted molecular inhibition, is therefore a highly desirable treatment strategy that may minimize complications arising from both tumor and treatment and more importantly be suitable for patients whose options are limited with respect to surgical or radiosurgical interventions. In this study we chose to examine the effect of Nilotinib on VS. Nilotinib (Tasigna®) is a second-generation receptor tyrosine kinase (RTK) inhibitor with a target profile similar to that of imatinib (Gleevec®), but increased potency, decreased toxicity and greater cellular and tissue penetration. Nilotinib targets not only the BCR-ABL oncoprotein, but also platelet-derived growth factor (PDGF) receptor signalling. In this preclinical study, the human *NF2*-null schwannoma cell line HEI-193 subjected to nilotinib inhibition demonstrated decreased viability, proliferation and anchorage-independent growth, and increased apoptosis. A daily dose of nilotinib for 5 days inhibited HEI-I93 proliferation at a clinically-relevant concentration in a dose-dependent manner (IC_50_ 3–5 µmol/L) in PDGF-stimulated cells. These anti-tumorigenic effects of nilotinib were correlated to inhibited activation of PDGFR-α and PDGFR-β and major downstream signalling pathways. These experiments support a therapeutic potential for Nilotinib in VS.

## Introduction

Vestibular schwannomas (VS) are typically slow-growing tumors that arise from the Schwann cell sheath of the vestibulocochlear nerve. Though benign, they can cause significant morbidity through compression of neurologic structures and obstruction of cerebrospinal fluid flow. Effects on the vestibulocochlear nerve result in permanent hearing loss, tinnitus and disequilibrium, and facial nerve compression can cause facial weakness or paralysis and disfiguration. Symptoms leading to diagnosis occur in 0.7–1 per 100 000 and are generally unilateral, although in 5–10% of cases tumors are bilateral and associated with the cancer predisposition syndrome neurofibromatosis type 2 (NF2). Management options include observation, surgical excision and radiosurgery, all of which have significant short- and long-term adverse effects [1]–[3]. However, for a distinct population of NF2 patients, surgery and radiosurgery are not feasible and no additional therapy is currently available.

The tumor suppressor gene *NF2*, located on chromosome 22 at 22 q12.2, encodes the protein merlin [4], also called schwannomin [5], an ezrin, moesin and radixin (ERM) family protein that associates the actin cytoskeleton with the cell membrane [6]. Schwann cell-specific knockout of merlin in mice leads to initiation of schwannomas [7], likely through loss of inhibition of the AKT [8] and ERK1/2 [9] signalling pathways. These mediators of survival and proliferation are activated by cell surface receptors such as stem cell factor receptor (c-KIT) and platelet-derived growth factor (PDGF) receptor-α and -β, which are over-expressed and over-activated in sporadic and NF2-associated peripheral and vestibular schwannomas [10].

These *in vitro* studies using the immortalized *NF2*-null VS cell line HEI-193 [10] demonstrated that the receptor tyrosine kinase (RTK) inhibitor imatinib mesylate (Gleevec, STI571) decreased the activation of c-KIT, PDGFR-α and PDGFR-β, as well as ERK-1/2, AKT and FAK. Furthermore, imatinib treatment increased apoptosis and cell cycle arrest, while inhibiting cell growth and proliferation and decreasing anchorage-independent growth. While developed as an inhibitor of the kinase activity of the BCR-ABL oncoprotein in chronic myelogenous leukemia (CML) [11], [12], the efficacy of imatinib against other RTKs has led to its application as a therapy for several human cancers, including myeloid malignancies [13] and gastrointestinal stromal tumors [14]. The emergence of imatinib-resistant kinase mutants, however, warranted the development of new RTK inhibitors. The compound nilotinib (Tasigna, AMN107) was rationally designed to improve potency and selectivity over imatinib towards the BCR-ABL kinase [15]. Nilotinib is also effective against c-KIT, PDGFR-α, PDGFR-β, and discoidin domain receptor (DDR) −1 and −2 [16], but has a better toxicity profile [17] and greater cellular and tissue penetration [18] than imatinib, this latter consideration of particular significance for chemotherapeutic agents that must penetrate the blood-brain barrier. Nilotinib is currently in clinical use for treatment of imatinib-resistant CML and Philadelphia chromosome-positive acute lymphoblastic leukemia.

In the current study, we chose to investigate the in vitro therapeutic efficacy of nilotinib in the immortalized human NF2-null VS cell line HEI-193 in two different growth conditions: in Schwann cell growth medium (GM) or in media supplemented with PDGF-BB. We demonstrate that activation of PDGFR-α and PDGFR-β was inhibited, as well as their downstream mediators AKT and mTOR. Functional assays demonstrated decreased viability, proliferation and anchorage-independent growth, and increased apoptosis, in a dose-dependent manner. Taken together these results suggest that nilotinib may be a viable option for treatment of patients with vestibular schwannoma.

## Materials and Methods

### Nilotinib Treatment

HEI-193 is an HPV E6-E7 immortalized human vestibular schwannoma cell line (gift from Marco Giovannini and David Lim: Department of Cell and Molecular Biology, House Ear Institute, Los Angeles, CA), established after they obtained informed patient consent [19]. For cell viability, proliferation and apoptosis experiments, HEI-193 cells were plated at a density of 1×10^3^ cells/well in 96-well plates and grown for 24 hours in DMEM supplemented with 10% FBS. Cultures were then incubated with vehicle or nilotinib (Novartis, Switzerland) at concentration 0, 1, 3, 5, 10 or 20 µM for four days in the presence of one of two growth media: 100 ng/mL PDGF-BB (Cell Signaling) in DMEM, or Schwann cell growth medium (GM; [9]) consisting of DMEM, 10% FCS, 0.5 µM forskolin (Sigma), 10 nM β-heregulin, 0.5 mM 3-isobutyl-1-methylxanthine (IBMX; Sigma), and 2.5 µg/mL insulin (Sigma). Media containing nilotinib was refreshed daily, extrapolating from the 18-hour half-life of nilotinib in human serum. Functional assays were carried out on the fifth day as described below.

### Viability Assays

HEI-193 cells were plated, grown and incubated with nilotinib as described above. Trypan blue based viable cell counting was done every day for 5 days, or on the fifth day of nilotinib treatment, by using the Vi-CELL Coulter Analyzer (Beckman). The percentage of viable cells is plotted against each dose level and normalized to HEI-193 cells in PDGF-BB or GM with vehicle only. Cell viability was also evaluated by the 3-(4,5-dimethyl-thiazol-2yl)-5-(3-carboxymethoxyphenyl)-2-(4-sulfophenyl)-2H-tetrazolium (MTS) assay (CellTiter 96 AQueous Assay System; Promega) as well as CellTiter-Fluor™ CelViability Assay (Promega) according to the manufacturer's instructions. MTS labelling reagent was added on the fifth day of nilotinib inhibition, and the absorbance at 490 nm determined following four hours of incubation using plate reader (Versa max, Molecular Devices). CellTiter-Fluor reagent was added for 3 hours on the fifth day of nilotinib inhibition, and the fluorescence was measured according to the manufacturing instructions. The mean of six experimental replicates from three independent experiments was determined for each nilotinib concentration. Statistical analysis was carried out using Student’s t-test.

### Proliferation Assay

HEI-193 cells were grown and incubated with nilotinib as described above. On the fourth day of nilotinib inhibition, bromodeoxyuridine (BrdU) was added to cell cultures and incubated for the final 18 hours of growth. Cells were fixed and the ELISA carried out according to the manufacturer’s instructions (Roche, Switzerland). Sample absorbance was measured at 450 nm. The mean of six experimental replicates from three independent experiments was determined for each nilotinib concentration. Statistical analysis was carried out using Student's t-test.

### Apoptosis Assay

HEI-193 cells were plated, grown and incubated with nilotinib as described above. Apoptosis was evaluated using the Apo-One Homogeneous Caspase-3/7 Assay (Promega), with addition of Apo-One reagent at 5 days following the final dose of nilotinib. The reagent was incubated for 6 hours and fluorescence measured according to the manufacturer’s instructions (485 nm excitation/527 nm emission). The mean of six experimental replicates from three independent experiments was determined for each nilotinib concentration. Statistical analysis was carried out using Student's t-test.

### Cell Cycle Analysis

HEI-193 cells were grown in DMEM supplemented with 10% FBS for 2 days, serum-starved for 24 hours and then inhibited with nilotinib (0, 3, 5, 10, or 20 µM) in PDGF-BB or GM for 2 days. Cell cycle analysis was carried out using propidium iodide (PI; BD Pharmingen) staining and fluorescence-activated cell sorting (FACScan; Becton Dickinson). Cells were fixed with 80% ethanol for 18 hours at 4°C, centrifuged at 1500 rpm, resuspended in solution containing PI (0.1 mg/mL) and RNaseA (2 mg/mL), and incubated for 40 minutes at room temperature. Following repeat centrifugation, cells were resuspended in 500 µL PBS for FACS analysis. Samples were analyzed using CellQuest Pro (Becton Dickinson) software. Sub G_1,_ G_1_, S, G2/M-phase fractions were calculated as the mean of duplicate experiments.

### Soft Agar Clonogenic Assay

Growth media containing 0.5% agar was added to the bottom of each 60 mm well. HEI-193 cells (1.0×10^4^) were suspended in a mixture of 0.35% low-melting point agar (Cambrex), RPMI 1640 and 10% FBS, and plated over solidified base agar. Either PDGF-BB medium or GM containing vehicle or nilotinib (5, 10 or 20 µM) was added to the agar surface, and changed 5 times per week. Plates were incubated at 37°C for 3 weeks. Colonies growing in the agar were stained with 0.005% crystal violet (Sigma) and visualised using AxioVision (Carl Zeiss, Canada) on an inverted microscope. Clusters larger than 500 µm were counted as discrete colonies, and the maximal diameter of colonies was recorded. The soft agar assay was performed in triplicate, with two independent experiments analyzed by Student's t-test.

### Western Blot Analysis

HEI-193 cells grown in DMEM with 10% FBS to 50% confluence, then serum-starved for 24 hours. Cells were treated with vehicle or nilotinib (3, 5, 10 or 20 µM) for 30 minutes or 24 hours, followed by a 10-minute incubation in PDGF-BB medium or GM in the presence or absence of nilotinib.

Ras-GTP was extracted by applying 600 µg of protein to glutathione S-transferase-Ras-binding domain of Raf1 (GST-RBD; Ras Activation Assay Kit, Upstate Biotech). In brief, cells were lysed in Mg^2+^ buffer containing protease (Roche) and phosphatase (Calbiochem) inhibitors and then incubated for 1 hour at 4°C with glutathione-sepharose beads bound to 6.0 µg of GST-RBD. The beads were then washed in lysis buffer, followed by immunoblotting of the supernatant with the anti-Ras antibody (1∶2000; Upstate Biotech).

Immunoblotting of activated receptor and other downstream mediators was carried out on whole-cell lysate. In brief, cells inhibited and stimulated as described above were lysed on ice in modified PLC lysis buffer supplemented with protease (Roche) and phosphatase (Calbiochem) inhibitors, and protein concentration determined using BCA assay. Equal quantities of whole-cell lysate were separated on 7% SDS-PAGE gel and transferred to PVDF membrane (NEN) using a semi-dry transfer apparatus (Bio-Rad). Membranes were probed overnight with activation-specific phosphotyrosine antibodies to PDGFR-α (Tyr754, Abcam; 1∶500), PDGFR-β (Tyr771, Cell Signaling; 1∶250), AKT (Ser473, Cell Signaling; 1∶1000), ERK1/2 (Thr202/Tyr204, Cell Signaling; 1∶1000), S6 ribosomal protein (Ser240/244, Cell Signaling; 1∶1000), and mTOR (Ser2448, Cell Signaling; 1∶1000). Total protein expression was determined using non–activation-specific antibodies: PDGFR-α (Cell Signaling; 1∶400), PDGFR-β (Cell Signaling; 1∶400), AKT (Cell Signaling; 1∶1000), ERK1/2 (Cell Signaling; 1∶1000), S6 ribosomal protein (Cell Signaling; 1∶1000), mTOR (Cell Signaling; 1∶1000) and β-actin (Sigma; 1∶20,000). Horseradish peroxidase–conjugated secondary antibody (Bio-Rad) was used, with visualization carried out using Chemiluminescence Reagent Plus (Perkin-Elmer). Densitometric analysis was undertaken to determine the ratio of phosphorylated-to-total protein levels. The mean ratio of phosphorylated/total protein as a function of nilotinib dose was analyzed by Student’s t-test. All densitometric analyses were undertaken within the linear range with the AlphaEaseFC software. Fold changes were calculated using the vehicle-containing PDGF- or GM-stimulated treatment as a standard value of 1.

## Results

### Optimal Inhibition Conditions

**Figure 1:**
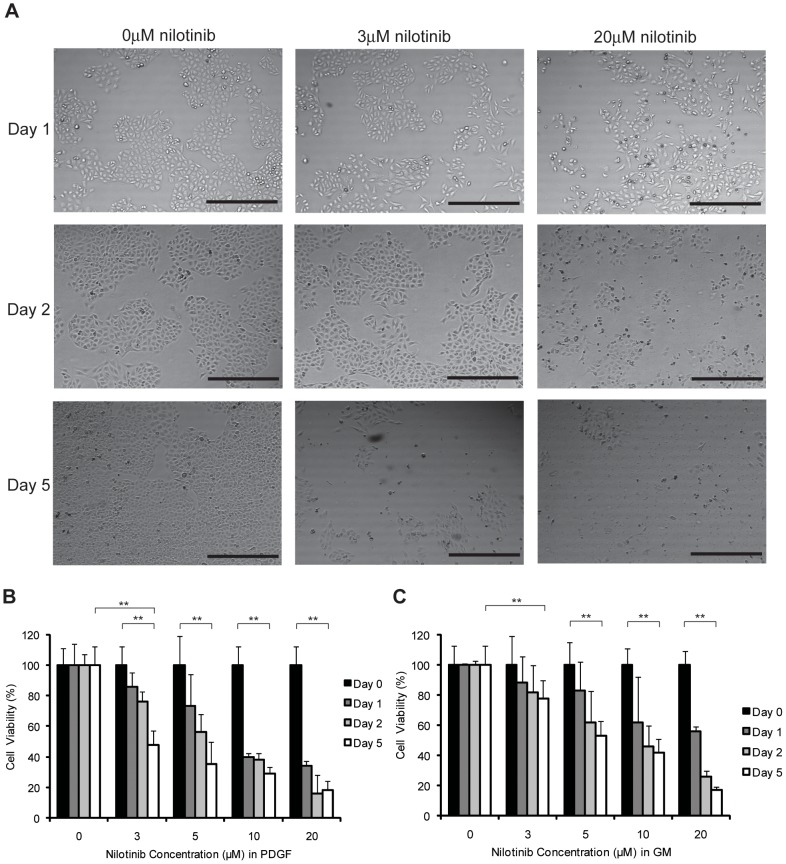
Nilotinib decreases HEI-193 cell viability in a time-, dose-, and growth condition- dependent manner. (A) HEI-193 grown in (PDGF or GM) were treated with 0, 3 or 20 µM nilotinib. Phase contrast microscopy images obtained at 1, 2 and 5 days show fewer cells with increasing nilotinib concentration. Scale bar  = 500 µm. (B) HEI-193 grown in PDGF media were treated with 0, 3, 5, 10 or 20 µM nilotinib up to 5 days. Trypan blue viability assay shows decreased number of viable cells at 5 days in 3 µM nilotinib, compared to vehicle shown, mean and SD of six technical replicates. **denotes significant (p<0.005) difference between treated and untreated samples. (C) HEI-193 grown in GM were treated with 0, 3, 5 or 20 µM nilotinib up to 5 days. Trypan blue viability assay shows decreased number of viable cells at 5 days in 5 µM nilotinib. Shown, mean and SD of six technical replicates. **denotes significant (p<0.005) difference between treated and untreated samples.

Initial experiments were carried out to establish concentration of nilotinib which is most tolerated by cells when exposed for longer duration (Fig. 1). Our results show that 3 µM concentration of nilotinib was tolerated by cells for at least 5 days before a significant reduction in viability was observed (1b), and 5 µM for cells grown in GM. This concentration of nilotinib also happens to be clinically relevant, below the mean peak inhibitor concentration (4.27 µM) found in the serum of patients treated for CML [18].

### Reduction in Cell Viability and Proliferation

**Figure 2:**
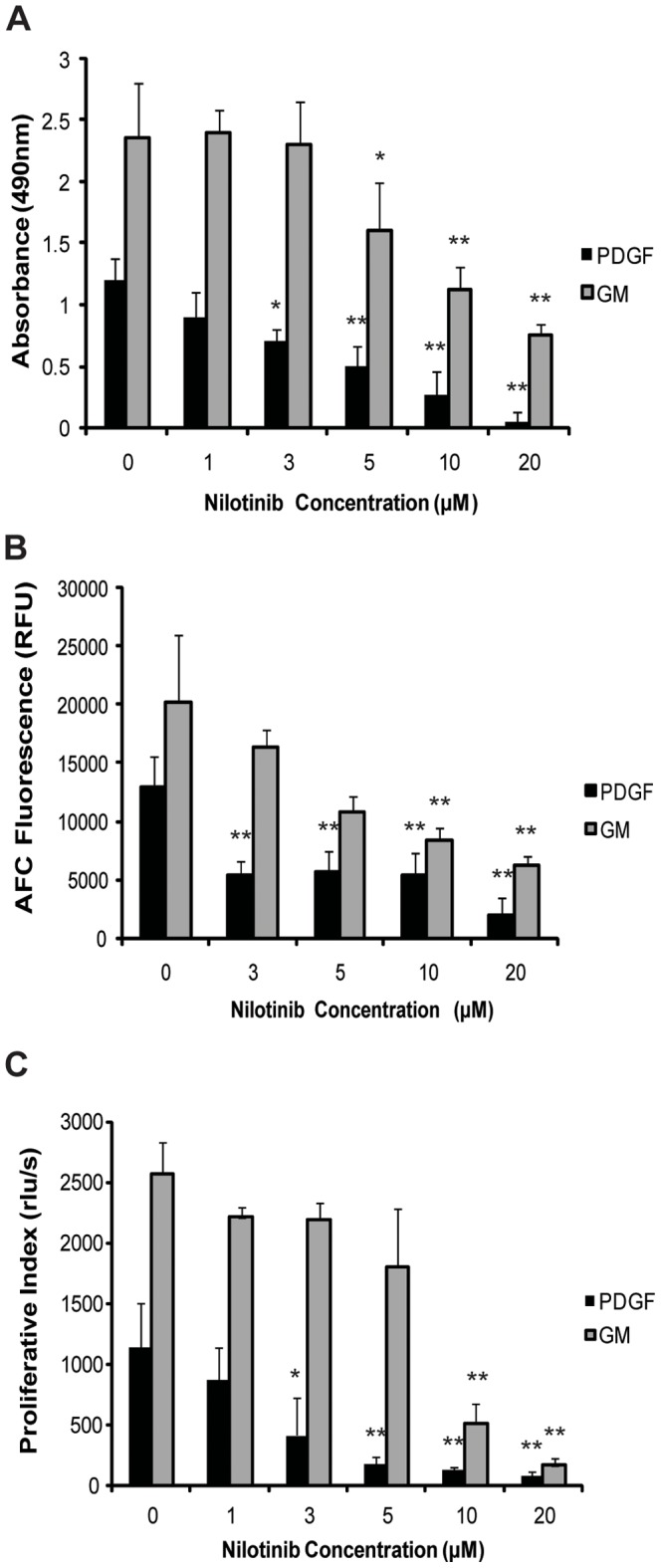
Nilotinib decreases viability and proliferation of HEI-193 cells in a dose-dependent manner. HEI-193 cells grown in PDGF-BB or GM were evaluated after 5 days of treatment with 0, 3, 5, 10 or 20 µM nilotinib. Shown, mean value of six technical replicates. *denotes significant value (*p*<0.05), **denotes significant value (p<0.005) (A) Nilotinib decreased absorbance on MTS viability assay in both growth conditions. (B) Compared to cells grown in vehicle control, nilotinib reduced the absorbance on fluorescence viability assay in both growth conditions. (*c*) Proliferation measured by BrdU incorporation was decreased by nilotinib in both growth conditions.

HEI-193 cells grown either in media supplemented with PDGF-BB or in GM were administered daily doses of nilotinib. The lowest concentration of nilotinib which resulted in significant reduction in viable cells for PDGF-BB condition after 5 days treatment, was determined to be 3 µM, by two separate assays, MTS (Fig. 2A) and fluorescent based viability assay (Fig. 2B). Higher concentrations did not provide additional advantage in terms of decreased cell viability except very high (20 µM) concentration of nilotinib.

Decrease in cell viability is also reflected by significant and concomitant decrease in proliferating cells upon treatment with 3 µM nilotinib for 5 days (Fig. 2C). For the cell cultured in GM conditions, a higher nilotinib concentration (5 µM) is effective for significant decrease in cell viability (as shown by two separate assays; Fig. 2A and B) and proliferation (Fig. 2C). A further decrease in viability and proliferation was observed at higher concentrations of nilotinib.

As shown in Fig. 1A and B, cells cultured in PDGF-BB conditions and exposed to 3 µM concentrations of nilotinib was well tolerated for longer time as compared to 20 µM, as well as 5 µM for cells grown in GM (data not shown and Fig. 1c). Nilotinib thus decreased cell viability and proliferation in a manner that is dependent upon drug concentration as well as growth conditions.

### Increase in Caspase-dependent Apoptosis

**Figure 3:**
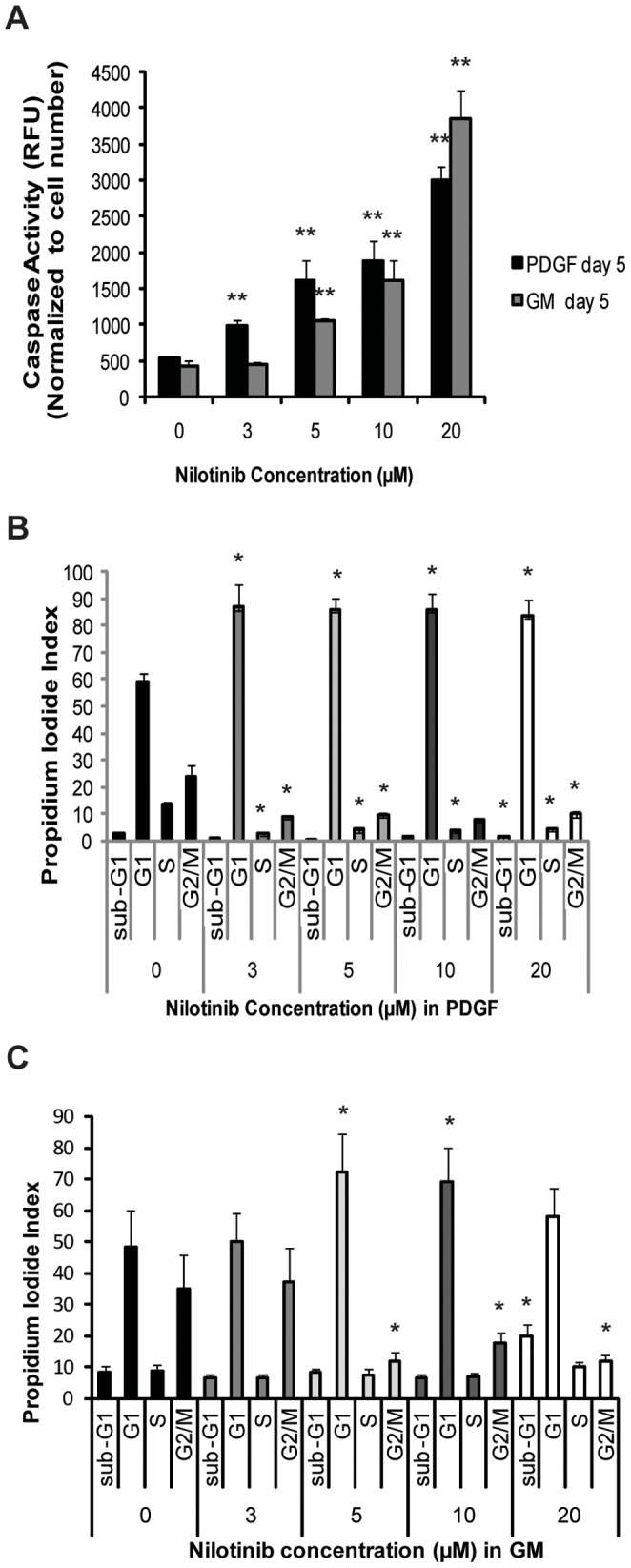
Nilotinib induces apoptosis and cell cycle arrest in HEI-193 cells at clinically-relevant concentrations. (A) HEI-193 cells were treated with 0, 3, 5, 10 or 20 µM nilotinib for 5 days. In PDGF-BB, an increase in caspase-3/7 activity is seen at ≥3 µM; in GM, caspase-3/7 activity is increased at ≥5 µM. Fluorescence signal normalized to number of viable cells. Shown, mean value of six technical replicates. (B) HEI-193 cells were treated with 0, 3, 5, 10 or 20 µM nilotinib for 2 days. In PDGF-BB-supplemented media, the proportion of cells in G1-phase is increased, and the proportion in S- and G2/M-phase decreased, at all concentrations tested. Shown, mean of two replicate experiments. (C) In GM, the proportion of cells in G1-phase is increased and the proportion in G2/M decreased at ≥5 µM nilotinib. Shown, mean of two replicate experiments.

As determined by caspase-3/7 activity assay normalized ratio to cell number, nilotinib induced apoptosis in HEI-193 in a dose-dependent manner. In cultures grown in media containing PDGF-BB, in the presence of ≥3 µM nilotinib, caspase-3/7 activity was significantly increased (*p*<0.005) at 5 days following the final inhibitor dose (Fig. 3*a*). When cells grown in GM were assayed for caspase-3/7 activity at 5 days following the final nilotinib dose, an increase in apoptosis (*p*<0.005) was observed at ≥5 µM nilotinib compared to vehicle (Fig. *3a* *)*.

### Induction of Cell Cycle Arrest

To explore the cell cycle status of cells treated with nilotinib, concentration and duration of exposure was selected where control and treated cells were phenotypically comparable (3 µM for 2 days in PDGF and 5 µM for 2 days in GM conditions). Measurement of propidium iodide (PI) staining demonstrated a significant increase (*p*<0.05) in the number of cells in G1 phase following 2 days of nilotinib treatment (Fig. 3*b*). For cells grown in PDGF-BB, this increase was observed in the presence of ≥3 µM nilotinib with no further increase at higher concentrations. This increase in G1 was accompanied by a reduction in the number of cells in S-phase, as well as reduction in the G2/M cycle. An increase in the number of cells in G1 phase, with a corresponding reduction in G2/M-phase cells, was observed in the presence of ≥5 µM nilotinib for cells grown in GM (Fig. 3c), with not statistically changes in the S-phase.

### Reduction in Anchorage-independent Growth

**Figure 4:**
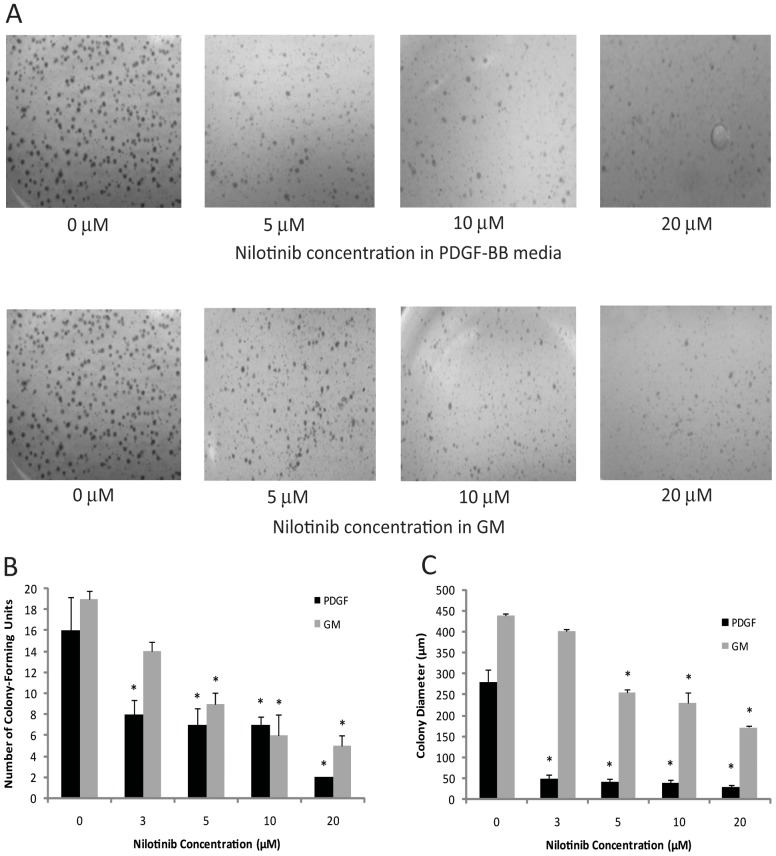
Nilotinib decreases the *in vitro* tumorigenicity of HEI-193 cells. Anchorage-independent growth in soft agar was inhibited in the presence of nilotinib. (A) Visual assessment of agar plates shows qualitative decrease of colony formation by nilotinib. (B) The number of colonies was reduced at ≥3 µM nilotinib in media containing PDGF-BB, and at ≥5 µM in GM. *denotes significant value (*p*<0.05). (C) The mean maximal colony diameter was decreased by nilotinib at ≥3 µM in PDGF-BB media, and at ≥5 µM in GM. *denotes significant value (*p*<0.05).

HEI-193 cells treated with nilotinib showed a dose-dependent decrease in the number and size of colonies formed in soft agar (Fig. 4*a*). Cells grown in PDGF-BB formed fewer (Fig. 4*b*) and smaller (Fig. 4*c*) colonies in ≥3 µM nilotinib, compared to vehicle. In GM, the presence of ≥5 µM nilotinib reduced the total number (Fig. 4*b*) of colonies formed, as well as their diameter (Fig. 4*c*).

### Reduced Activation of Targeted Receptors

**Figure 5:**
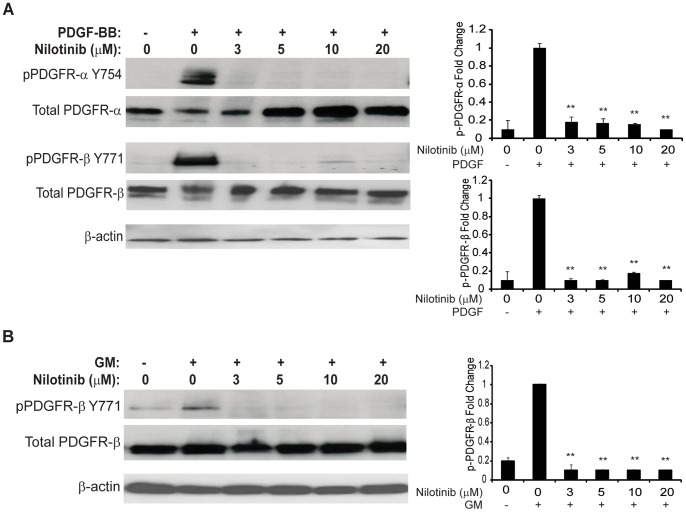
Nilotinib inhibition of HEI-193 cells decreases activation of PDGFR-α and PDGFR-β. HEI-193 cells inhibited with nilotinib and stimulated with PDGF-BB or GM were lysed and analyzed by Western immunoblot using phophorylation-specific antibodies. Comparison was made to total protein expression, as determined by immunoblotting using non-activation specific antibodies. Densitometry was used to evaluate fold-change in phosphorylation, with values normalized to activation in the presence of vehicle control. **denotes significant (*p*<0.05) difference compared to phosphorylation in vehicle control. (A) PDGF-BB activation of both PDGFR-α and PDGFR-β is abrogated in the presence of ≥3 µM nilotinib. (B) GM-stimulated activation of PDGFR-β is inhibited by ≥3 µM nilotinib.

HEI-193 cells were pre-treated for 30 minutes with nilotinib of different concentrations prior to stimulation with PDGF-BB or GM. Quantification of phosphorylation-specific immunoblot assays, normalized to total receptor expression, showed that PDGF-BB stimulation for 10 minutes resulted in high activation of the PDGFR-α and PDGFR-β receptors (Fig. 5a). Stimulation with GM activated PDGFR-β (Fig. 5b); however, phosphorylation of PDGFR-α did not increase above baseline (data not shown). A significant decrease in receptor activation was seen with both PDGF-BB and GM stimulation at nilotinib concentration as low as 3 µM. The expression for total PDGFR-α and PDGFR-β receptors decreased upon stimulation with PDGF-BB, likely due to rapid receptor endocytosis kinetics upon ligand binding.

### Inhibition of Downstream Mediators

**Figure 6:**
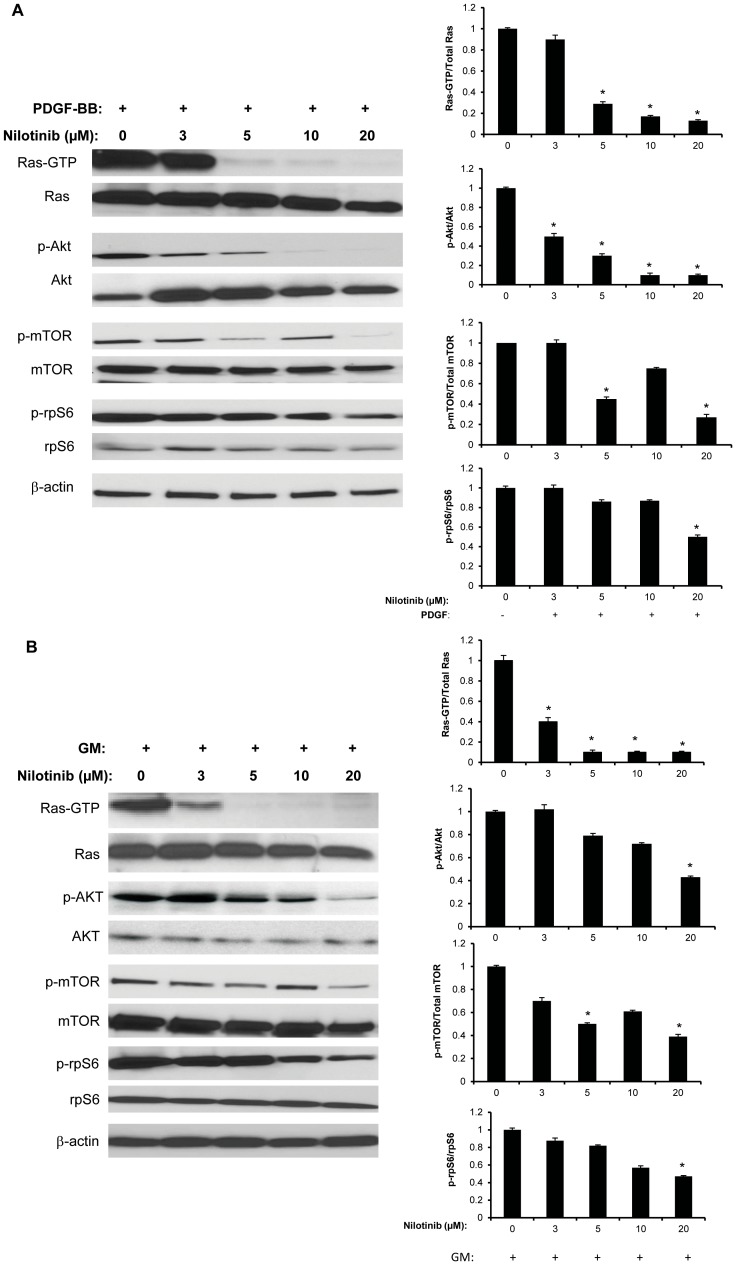
Nilotinib inhibition of HEI-193 decreases signaling downstream of PDGFR. HEI-193 cells inhibited with nilotinib and stimulated with PDGF-BB or GM were lysed and analyzed by Western immunoblot using phophorylation-specific antibodies. Comparison was made to total protein expression, as determined by immunoblotting using non-activation specific antibodies. Densitometry was used to evaluate fold-change in phosphorylation, with values normalized to activation in the presence of vehicle control. *denotes significant (*p*<0.05) difference compared to phosphorylation in vehicle control. (A) PDGF-BB-mediated activation of Ras and AKT are inhibited at low nilotinib concentration. Inhibition of S6 ribosomal protein phosphorylation is seen at high nilotinib concentration, and mTOR phosphorylation is variable with nilotinib concentration. (B) GM-mediated activation of Ras is inhibited at low nilotinib concentration. Inhibition of AKT and S6 ribosomal protein phosphorylation is seen at high nilotinib concentration, and mTOR phosphorylation is variable.

HEI-193 cells were pre-incubated with nilotinib for 24 hours then stimulated with PDGF-BB or GM for 10 minutes. The addition of either PDGF-BB or GM resulted in activation of effectors involved in multiple pro-tumorigenic pathways, including Ras, AKT, mTOR, and S6 ribosomal protein (Fig. *6a, b*). In PDGF-BB media, Nilotinib was effective, in inhibiting activation of AKT at 3 µM, of Ras and mTOR at 5 µM nilotinib. With the addition of GM, activation of Ras was inhibited at 3 µM nilotinib, and of mTOR at 5 µM nilotinib. Decrease of phosphorylation of AKT was seen at 20 µM nilotinib in GM. For both conditions, decrease in phosphorylation of S6 ribomsomal protein was seen at 20 uM of nilotinib and levels of mTOR activity fluctuates as nilotinib concentrations increases (*Fig. 6a*, b), (see discussion). Activation of ERK1/2 was not inhibited by nilotinib to any significant extent (data not shown).

## Discussion

Vestibular schwannomas are benign tumors that grow slowly and can often be managed with close observation. In instances of large or growing and symptomatic tumors, surgery or radiosurgery may be indicated, with potential complications and adverse effects. In addition, for a distinct population of NF2 patients, surgery and RT are not feasible and no additional therapy is currently available. Hence, there is a compelling need to identify efficacious and well-tolerated systemic therapy. Such therapy must have minimal adverse effects over the long term, and can ideally be administered at a low dose. The efficacy and utility of targeted tyrosine kinase inhibition was demonstrated by imatinib in the chronic treatment of CML driven by the BCR-ABL oncogene [11], [12]. Its sister compound nilotinib also targets BCR-ABL, as well as c-KIT and PDGFRs, and is an appealing candidate for management of VS due to its improved side-effect profile and greater cell and tissue penetration.

Previous studies have demonstrated increased expression and activation of RTKs, particularly PDGFR-β, in human schwannoma specimens and primary schwannoma cells [9], [20]. Nilotinib has been shown to reduce proliferation in primary schwannoma cell cultures at concentrations significantly lower than imatinib [20]. These are promising results, but examine PDGFR-β in isolation; as patient-derived schwannomas express and activate both PDGFR isoforms, the paradoxical decreased effect observed at higher nilotinib concentration could be due to increased activation of alternative receptors, such as PDGFR-α. Furthermore, the cell viability maintained at the very low nilotinib concentration (0.5 µM) examined in this study may lead to emergence of resistant mutants over time in a disease that requires life-long treatment.

In contrast, in our study, we aimed to assess the role of PDGFR-α in addition to PDGFR-β. We show that nilotinib administered continuously at 3 µM for five days reduces viability of HEI-193 cells, which results from reduction of proliferation and increase of cell cycle arrest and apoptosis. This prolonged treatment is critical to achieve continuous inhibition of the receptors and to determine whether resistant mutants will arise over time. In addition, nilotinib reduces the number and size of colonies formed in anchorage-independent growth assay. The observed reduction in cell viability and clonogenicity results from nilotinib inhibiting the PDGF-BB-mediated activation of PDGFR- α, PDGFR- β, AKT, and mTOR, which it effects at a concentration below the mean peak inhibitor concentration (4.27 µM) found in the serum of patients treated for CML [18]. Thus, by increasing apoptosis and reducing viability among cells of an established tumor, nilotinib may be able to reduce tumor mass with tolerable systemic toxicity. When cells are grown in GM, similar anti-tumorigenic effects are observed, but at slightly higher inhibitor concentration. This difference in sensitivity to nilotinib is likely due to activation of other receptors by factors released from the cells. As strong activation of PDGFR-α is observed only when cells are grown in PDGF-BB, and not in GM, the former better recapitulates the growth environment of schwannomas, which exhibit increased PDGFR-α expression and activation compared to Schwann cells [10].

While the prolonged treatment used in the viability assays demonstrated a sustained inhibition of PDGFR, a trend toward decreased cell viability was observed as early as 24 hours into treatment of cells cultured in PDGF-BB with 3 µM nilotinib, and a significant decrease at 5 days. This correlated with a significant increase in cell cycle arrest, as evidenced by accumulation of cells in G1 phase, observed two days after start of treatment with 3 µM nilotinib, as well as an increase in caspase-3/7 activity at 5 days of treatment. A similar pattern was observed for cells cultured in GM conditions (Fig. 1*c*).

The effect of nilotinib on receptor activation and tumorigenic behavior correlate with its inhibition of PDGF-BB- and GM-stimulated activation of downstream signaling mediators AKT and mTOR. These findings are in keeping with established evidence of the PI3K and mTOR pathways as critical regulators of schwannoma growth [21]. Inhibition of PDGFR-α and -β occurs at a low concentration of 3 µM which leads to inactivation of downstream signaling but at different nilotinib concentrations. While Ras activation is decreased at low nilotinib concentrations, there is no significant effect on ERK1/2 phosphorylation, which is in part explained by the presence of non-PDGFR-mediated means of activating ERK1/2, as previously described in schwannomas [9]. This supports the concept proposed by Ammoun *et al.* [20] that nilotinib may be beneficial as one component in combinatorial therapy. Phosphorylation of AKT is inhibited by 3 µM niltonib in PDGF-BB-stimulated cells, but mTOR activity fluctuates between 3 and 10 µM in both PDGF-BB and GM. This might be due to the regulation of mTOR activity not only at the PDGFR level but also by positive feedback mechanisms downstream of the receptor, such as saturation of PI3K activity [22]. Similarly, Ammoun *et al.* [20] showed that nilotinib at high concentration (10 µM) completely loses effect in down-regulating AKT and ERK1/2. Furthermore, decreased phosphorylation of S6 ribosomal protein is seen only at high nilotinib concentration, indicating the presence of alternative activation pathways further downstream.

One limitation of this study is the use of a single cell line for the investigations. The HEI-193 cell line was derived from a vestibular schwannoma in a NF2 patient, and immortalized with human papilloma E6–E7. A point mutation in NF2 results in a merlin mutant that has attenuated, but not absent, tumor suppressor activity [23]. Nevertheless, as HEI-193 cells have an expression and activation profile for RTK targets of nilotinib similar to human surgical VS specimens [10], it remains a valid model for the functional experiments carried out here and elsewhere. Similar to our work, Fraenzer *et al.* [24] stimulated HEI-193 cells with PDGF to study the effects of NF2 restoration on PDGFR degradation. Using the PDGF and GM stimulation strategy previously shown [9], [20], [24], we established the *in vivo* PDGFR receptor status in order to understand the mechanisms of nilotinib-mediated effect.

In summary, these *in vitro* results support the anti-tumorigenic activity of nilotinib in human vestibular schwannoma cells. These preclinical results provide the basis to support testing Nilotinib as potential biological therapy for growing VS. Given that there is demonstrated safety and tolerability of Nilotinib through extensive clinical experience with this compound in other tumor types, it would be safe to proceed with clinical studies testing the efficacy of Nilotinib in growing VS.
